# Comparative Study of Natural Terpenoid Precursors in Reactive Plasmas for Thin Film Deposition

**DOI:** 10.3390/molecules26164762

**Published:** 2021-08-06

**Authors:** Daniel S. Grant, Jakaria Ahmed, Jason D. Whittle, Andrew Michelmore, Krasimir Vasilev, Kateryna Bazaka, Mohan V. Jacob

**Affiliations:** 1 College of Science and Engineering, James Cook University, Townsville, QLD 4811, Australia; Daniel.Grant@my.jcu.edu.au (D.S.G.); Jakaria.Ahmed@my.jcu.edu.au (J.A.); katia.bazaka@anu.edu.au (K.B.); 2 UniSA STEM, University of South Australia, Mawson Lakes Campus, Adelaide, SA 5095, Australia; jason.whittle@unisa.edu.au (J.D.W.); andrew.michelmore@unisa.edu.au (A.M.); Krasimir.Vasilev@unisa.edu.au (K.V.); 3 School of Engineering, College of Engineering and Computer Science, The Australian National University, Canberra, ACT 2600, Australia

**Keywords:** plasma polymerisation, thin films, natural precursors, tea tree oil

## Abstract

If plasma polymer thin films are to be synthesised from sustainable and natural precursors of chemically heterogeneous composition, it is important to understand the extent to which this composition influences the mechanism of polymerisation. To this end, a well-studied monoterpene alcohol, terpinen-4-ol, has been targeted for a comparative study with the naturally occurring mix of terpenes (viz. *Melaleuca alternifolia* oil) from which it is commonly distilled. Positive ion mode mass spectra of both terpinen-4-ol and *M. alternifolia* oil showed a decrease in disparities between the type and abundance of cationic species formed in their respective plasma environments as applied plasma power was increased. Supplementary biological assay revealed the antibacterial action of both terpinen-4-ol and *M. alternifolia* derived coatings with respect to *S. aureus* bacteria, whilst cytocompatibility was demonstrated by comparable eukaryotic cell adhesion to both coatings. Elucidating the processes occurring within the reactive plasmas can enhance the economics of plasma polymer deposition by permitting use of the minimum power, time and precursor pre-processing required to control the extent of monomer fragmentation and fabricate a film of the desired thickness and functionality.

## 1. Introduction

The development of two-dimensional materials, including single and multi-layer thin films fabricated from polymers and their composites, is critical for the advancement of miniaturized devices spanning energy, optoelectronics, biomedical and space applications [1,2,3,4,5]. For these devices to function efficiently over extended periods of time, the quality of these films and the precision with which they are assembled need to be sufficiently high [6]. Yet, techniques that offer superior precision, such as atomic layer deposition, are difficult to scale up and lack affordability [7]. On the other hand, while more affordable, flexible and more amenable to integration into existing processes and scale up, printing of polymer films are yet to offer the needed level of precision. Other considerations include the related issues of quality of interlayer adhesion, compatibility of methods for multi-layer assembly and finding suitable pre- and post-treatment methods to enhance interlayer adhesion and surface properties [8,9].

Plasma polymerisation is a well-established technique for the fabrication of thin films from a wide range of monomer species, including volatile precursors, at low and atmospheric pressure [10,11,12]. The product of plasma polymerization is a result of a complex interplay between species within the plasma volume itself and those at the plasma-substrate interface [13,14]. The chemical and physical properties of these films are heavily dependent upon a number of process parameters, including precursor species, flow rate, applied power, pressure, and substrate temperature and surface topography [15,16,17]. By quantifying the role that each of these parameters play in influencing the nature and energies of plasma species, it is possible to precisely manipulate the deposition process in order to fabricate films exhibiting specific properties [18,19].

The drive for sustainability has spurred growing interest in replacing synthetic or highly purified organic precursors with natural, minimally-processed, and abundant low-cost alternatives [20]. One exemplar of this drive is the use of inherently volatile essential oils as precursors in plasma-assisted synthesis of polymer thin films and other carbon nanostructures [17,21,22,23]. Polyterpenol, a plasma polymer synthesised from the terpinen-4-ol precursor monomer, is among the best-studied essential oil-based plasma polymers. Terpinen-4-ol is a non-synthetic monocyclic terpene derived from the distillation of *M. alternifolia* oil (a.k.a. tea tree oil).

By virtue of the plasma polymerisation process, polyterpenol thin films share a number of attributes common to other plasma polymers including smooth, pinhole-free surfaces and optical transparency [24,25,26]. Beyond this, post-deposition retention of terpinen-4-ol functionality within biodegradable polyterpenol thin films fabricated at low power enables their use in biomedical and marine applications to retard bacterial fouling [27,28,29,30]. When fabricated at high input power, polyterpenol films demonstrate electron-blocking hole-transporting behavior, which can be used to direct the motion of carriers within organic optoelectronic devices [31,32].

As is the case for most high-tech thin film applications, there is a direct relationship between the consistency of nanoscale properties and subsequent material or device performance. In this respect, plasma polymers fabricated from essential oils rather than highly purified chemicals face two considerable challenges to their commercial uptake. First, there is chemical heterogeneity and batch variability of natural precursors, especially when entire oils rather than individual components are used. Yet, the use of minimally processes natural materials is preferred from the perspective of cost efficiency, environmental considerations and ease of processing. Secondly, plasma polymerization itself is an exceptionally complex process, owing to the diverse range of species present within the plasma and the extent to which interplay between these species is reliant upon deposition parameters. This poorly defined and complex chemistry can lead to the formation of films with variable structure and surface functionalities. Tuning of these parameters is typically performed using considerable empirical investigations.

In order to realise reproducible fabrication of quality high-value films from essential oils, it is imperative to understand the input chemistry and how it relates to the subsequent chemistry of the film. Hence, the focus of this work will be on the comparisons between terpinen-4-ol and *M. alternifolia* oil plasma environments at various powers so that it can be used for informed and cost-optimised decision-making with respect to the choice of precursor and plasma power for a specific application. Whilst the complexity of the plasma polymerisation process makes it difficult to arrive at this understanding, mass spectrometry (MS) has been employed to generate a mechanistic understanding of the plasma chemistry of a number of precursors with biomaterial applications, including acrylic acid, allylamine and ethanol [33,34,35].

## 2. Results and Discussion

MS was identified as a useful tool for understanding the processes occurring within the plasma volume following the introduction of a volatile plasma polymer precursor candidate. MS utilises an ion source, analyser and detector to generate a two-dimensional plot of signal intensity versus *m/z* (mass-to-charge ratio), with the product given as the mass spectrum. These spectra yield a wealth of information relating to chemical processes occurring within the plasma, including fragmentation, recombination, and the abundance of species involved in these reactions. As MS processes occur under high vacuum (resulting in long mean free paths), one can examine the gas phase chemistry of isolated ions that arrive at the grounded surface beneath the plasma sheath at the sampling entrance.

### 2.1. Plasma Characteristics—Commonly Reported Parameters

Table 1 outlines a number of key plasma and polymer parameters characterised throughout the course of this experiment. Specifically, it details nominal generator power (i.e., the output power from the R.F. generator), actual power coupled into the plasma (as measured with the OctIV Probe), terpinen-4-ol monomer flow rate into the reactor vessel, reactor vessel pressure and the mass rate at which the polymer was deposited (obtained via crystal quartz microbalance (QCM)).

It is common to report various plasma and thin film properties as a function of the nominal generator output power, which may be readily obtained from the display on the generator unit. Unfortunately, this figure ignores parasitic energy losses within the system, and thus inevitably over-represents the actual power delivered to the plasma. For this study, however, access to an OctIV Probe permitted quantification of the actual transmitted power. The disparity between the nominal generator power and the actual transmitted power can be attributed to various energy losses and inefficiencies inherent to the deposition assembly employed for this study. These inefficiencies include transmission line losses, matching network losses, and coupling losses between the electrodes and plasma volume [36].

The rate (Φ) at which the terpinen-4-ol monomer flowed into the reactor vessel was controlled by a needle valve, and is expressed in standard cubic centimeters per minute (sccm). By observing that the maximum deposition rate occurs for the minimum flow rate (i.e., at 0.7 sccm), we can speculate that the flow rate (and thus the number of monomeric units delivered to the plasma volume and available for fragmentation/deposition) is not a limiting factor in film formation. It must be noted, however, that this observation is only relevant if one discounts the influence of applied power on the deposition rate (which is also varied). Pressure exhibited an increasing trend in response to increases in the actual transmitted power, as one would expect following the associated increase in the density of energetic species within the plasma [18].

The Yasuda Factor given in Equation (1) (where *W* = wattage in J/s, *M* = monomer molecular weight in kg/mol, and *F* = monomer flow rate in mol/s) provides a measure of a system’s plasma energy density [37], and allows for a general comparison of plasmas operating at different conditions within the same reactor vessel [38].

*YF* = *W*/*MF* (1)

In this instance, *M* is held constant, and as such the *YF* can be reduced to an applied power-to-flow rate ratio (P/Φ) to facilitate comparison between the plasmas generated in this study. It must be noted when interrogating information presented in Table 1 (and subsequent figures) that it was not possible to undertake mass deposition rate and MS measurements at precisely the same P/Φ.

Deposition rates for plasma polymers derived from non-synthetic sources have been shown to demonstrate both a positive correlation with power (e.g., γ-terpinene [39]) and a negative correlation with power (e.g., linalool [40]). This deposition rate is highly dependent upon the energy invested per particle of gas mixture flowing through the glow discharge zone. For a given plasma reactor setup this is a function of several process parameters, including pressure, applied power, flow rate, and monomer species [13,16,18,19,41]. Beyond these parameters, it is known that substrate thermal and energetic conditions also influence plasma polymerisation processes, including adsorption, desorption, diffusion and chemical reactions [13,16,18,19,41]. These substrate conditions are themselves a function of the energy per bombarding particle and the flux density of these particles [18]. Hence, the reduction in mass deposition rate from 25 W to 50 W may indicate that an adsorption-desorption equilibrium is beginning to become a rate limiting process in polyterpenol deposition at higher powers.

**Figure 1:**
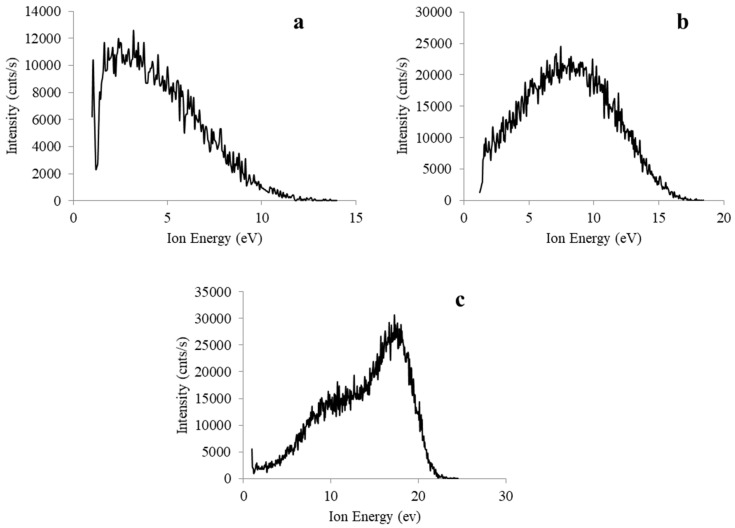
Positive ion energy distribution functions for *m/z* 154 and fragment ions arriving at the self-biased surface for powers and pressures of (**a**) 5 W 2.17 Pa, (**b**) 25 W 1.95 Pa, and (**c**) 50 W 1.69 Pa.

The decreasing deposition rate at 50 W may also be interpreted with respect to the ion energy distributions presented in Figure 1. Here we can assume that there is a shift in the balance from molecular physisorption, soft landing and chemisorption process (occurring between ~0.1–15 eV), to abstraction and sputtering processes at collision energies above ~15 eV [42,43]. It is also known that below a certain YF threshold, the rate of plasma polymerisation is strongly dependent upon the structure of the feed gas or monomer. At higher YFs the structure of the feed gas exerts less influence on the rate of deposition owing to increased monomer fragmentation [44].

Developing a macroscopic understanding of the role that the R.F. power plays in the deposition rate may permit further control over the plasma polymerisation process. Knowing the mass deposition rate under a particular deposition scheme (i.e., pressure, flow rate, etc.) also provides the means to selectively control polyterpenol film thickness by selecting an appropriate deposition time. Furthermore, the deposition rate may now prospectively be used as a metric for providing generalisations about the process conditions that exist within the plasma system.

### 2.2. Ion Energy Distribution Functions

The ion energy distribution function (IEDF) for positive ions arriving at the substrate is influenced by a number of parameters, including system pressure and instantaneous local electric field at the substrate [45]. Given that ion energy is not mass dependent, the IEDFs presented below are representative of all positive ions present within the plasma (not just those appearing at *m/z* 154).

The lack of a narrow ion energy band within Figure 1a–c can be attributed to pressure (or more precisely, the ratio of the mean free path of the ions to the sheath thickness). In this instance, increases in pressure lead to an increase in the prevalence of collisional processes in the sheath, which in turn serve to bring about a broadening of the IEDFs and a decrease in the mean energy [46,47]. Conversely, we can attribute the narrowing of the IEDFs as the transmitted power is reduced to ion transit time effects related to the extent of precursor monomer fragmentation. Specifically, at lower powers the monomer units exhibit minimal fragmentation (relative to higher powers), and these heavier ion fragments are not accelerated as rapidly as light ions. This, in turn, contributes to longer transit times and an associated reduction in the width of the IEDF [48].

Within non-thermal low pressure R.F. plasmas, the maximum ion energy is given by e_0_V_*sh*_, where e_0_ is the electron charge and V*_sh_* is the electric potential drop across the sheath in front of the substrate [49]. Thus, allowing for the electron charge to remain constant, the maximum ion energy is determined by the voltage drop across the substrate’s plasma sheath, *V_sh_*, [50], given as the sum of plasma potential *V_pl_* (assumed to be positive) and substrate potential *V_s_* (assumed to be negative):

*V_sh_* = *V_pl_* + *V_s_* (2)

The maximum ion energies given in Figure 1a–c indicate an increase in the maximum ion energy of the IEDFs as the generator and transmitted R.F. power is increased, a finding that is in keeping with those reported by other studies [51]. This relationship can be interpreted with respect to e_0_V_*sh*_ and Equation (2) by asserting that the increases in R.F. power serve to produce an increase in the sheath potential. Furthermore, given that the substrate is grounded (ensuring that *V_s_* = 0), these increases in sheath potential can be solely attributed to an increase in the plasma potential, *V_pl_*.

It is well known that the kinetic energy of bombarding ions plays a significant role in modifying the properties of thin films [16,18,19]. Specifically, processes such as ion implantation, etching, and chain-scissioning/cross-linking can result in variations in the density, adhesion, hardness and conductivity of plasma polymer thin films. For this reason, it is common for many plasma systems to employ a capacitively coupled R.F. biased substrate electrode to control the kinetic energy of the ions bombarding the substrate [16,18,19]. In this instance, however, our findings indicate that we can control this kinetic energy simply by careful selection of the generator power. This, in turn, may play an important role in controlling processes that are sensitive to energetic ion bombardment including etching rates, selectivity of the etched material and material degradation [29,45].

### 2.3. Plasma Characteristics—Residual Gas Analysis of Terpinen-4-ol

**Figure 2:**
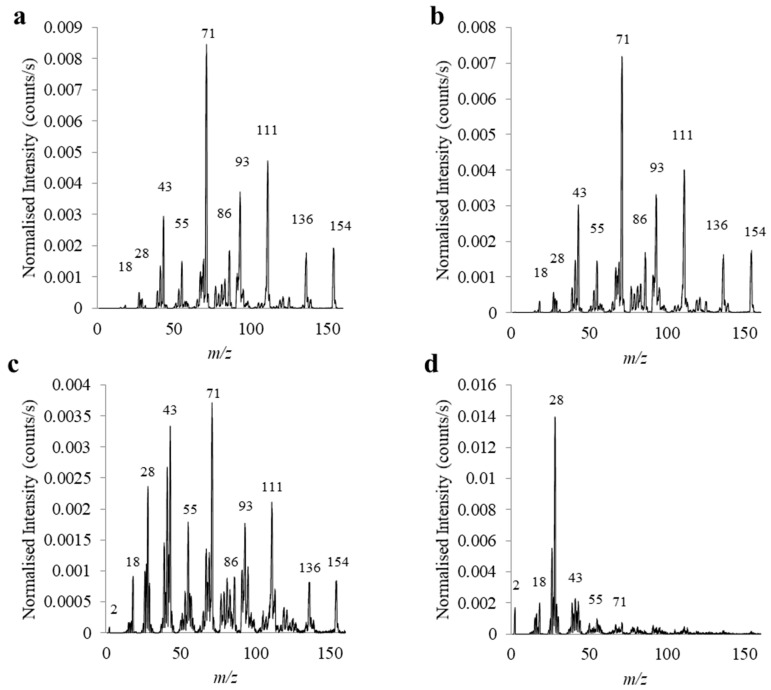
RGA mass spectra for (**a**) monomer with no plasma; and plasma-phase neutrals at (**b**) 5 W, (**c**) 25 W, and (**d**) 50 W.

Figure 2 depicts the electron impact ionisation residual gas analysis (RGA) mass spectra of the neutral terpinen-4-ol species, and *m/z* ratios present at R.F. powers of 5, 25 and 50 W. The spectra have been corrected to account for the instrument’s transmission function by assuming that peak intensity was proportional to m^−1^, as advised by the manufacturer. All spectra were also normalised with respect to the total area under the spectra peaks.

The *no plasma* spectrum was analysed by direct interpretation of signal peaks for ions produced by impact ionisation of the neutral species. Here, each peak represents a linear summation of all fragment ions with the corresponding *m/z* ratio, and it is probable that each such *m/z* relates to only a single fragment species (given that analysis is only being carried out on a single monomer and not a mixture of differing monomer species).

For *no plasma* the base peak is located at *m/z* 71, and additional molecular weight species are present at *m/z* 154 (representing the terpinen-4-ol monomer, either with or without scissioning), 136, 111, 93, 71 and 43. In this instance C_10_H_16_ is liable to degrade into *m/z* 93 with a loss of forty-three mass units (corresponding to C_3_H_7_), as evidenced by the comparable abundance of the complementary *m/z* 93 and 43 units. Increases in power resulted in a noticeable shift in intensities towards lower *m/z* species, including the detectable formation of H_2_ as evidenced by *m/z* 2.

RGA of the plasma-phase neutrals suggests extensive fragmentation of terpinen-4-ol. However, the mode of fragmentation at low power (i.e., at 5 W) is similar to that provided by electron impact in the *no plasma* sample, indicating that this power stimulates minimal plasma-induced fragmentation of the monomer. It is interesting to note that even at an applied power of 25 W (and to a lesser extent, 50 W), there remains a non-trivial source of precursor monomer within the plasma volume. This also indicates that some of the spectral intensity of *m/z* ratios less than that of the monomer may be wholly or partially attributed to the RGA electron impact fragmentation of these precursor monomers, as opposed to plasma-induced fragmentation. This stands in contrast to similar studies conducted on lower molecular weight monomers such as acrylic acid, where virtually total monomer fragmentation was achieved at 15 W. This disparity between spectral intensity of the monomer ions from an acyclic structure (such as acrylic acid) and the cyclic terpenin-4-ol may be partially accounted for by scissioning of the cyclic structure, which would result in a change in the ion’s molecular structure, but not its molecular weight. However, the relatively low intensity of *m/z* 154 peaks in (b–d) suggests that such contributions are likely to be of a modest nature. The remaining peaks can be attributed to plasma-induced fragmentation of the terpinen-4-ol monomer, with the resulting neutral fragments being ionised by the mass spectrometer.

At low YF we observe minimal fragmentation and considerable preservation of monomeric units. By assuming that these relatively intact monomeric units are incorporated into the plasma polymer, we can further substantiate findings presented in [23,27,28], which attribute the enhanced antibacterial properties of polyterpenol thin films fabricated at low YF to the preservation of monomer functionalities (i.e., -OH and double bonds) within the polymer film. Incorporation of monomeric units into the final deposit may be further advanced by increased adsorption of non-fragmented units at low substrate temperatures eventuating as a natural by-product of the low applied power [23,27,28]. Polymer films fabricated in this manner combine favorable surface properties, such as moderate hydrophilicity and micro- and nano-scale roughness that retards microbial attachment, with elution of biologically active agents that target microorganisms that manage to attach [22,23,27,52]. The biologically active agents that are released from the surface during biodegradation are thought to include monomer units as well as their derivatives, where the latter may enhance the biological activity of the coating by targeting different cellular components and processes to the monomer.

This finding permits us to tune the plasma parameters, and, in particular, the plasma power, in order to achieve the desired fragmentation scheme and subsequent incorporation of specific fragment (Table 2) and monomer species within the plasma volume and resulting plasma polymer deposit.

### 2.4. Plasma Characteristics—Positive Ion Mode Analysis of Terpinen-4-ol and M. alternifolia Oil

**Figure 3:**
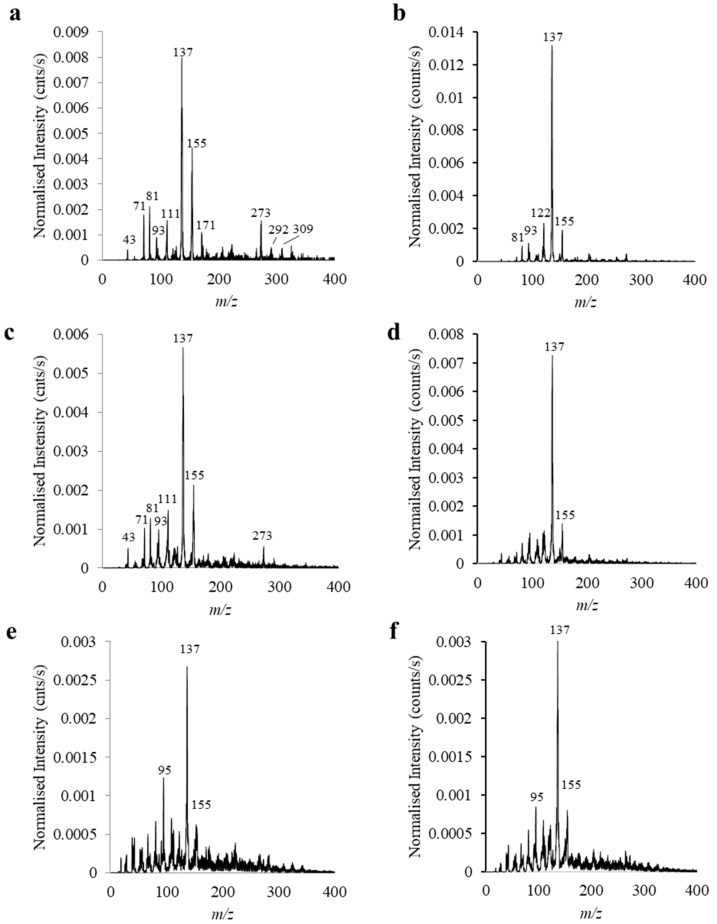
PIM mass spectra for terpinen-4-ol at R.F. plasma powers of (**a**) 5 W, (**c**) 25 W, and (**e**) 50 W, and the parent *M. alternifolia* oil at R.F. plasma powers of (**b**) 5 W, (**d**) 25 W, and (**f**) 50 W.

As detailed in Table 3, the monomer of interest in this study, terpinen-4-ol, is the primary subcomponent of *M. alternifolia* oil. Subsequently, a comparative analysis of the positive ion mode (PIM) mass spectra for the cationic species generated by the plasma environment alone (without any further electron impact ionisation-induced fragmentation) was performed for both the monomer and the parent oil, as shown in Figure 3. Associated peak assignments for terpinen-4-ol fragments are provided in Table 4.

Elimination of water (18 amu) from the monomer gives rise to the base peak at *m/z* 137 for all terpinen-4-ol spectra. Other features of note in the monomer spectra include the low intensity presence of a dimer at *m/z* 309, with a concomitant peak at *m/z* 292 following elimination of OH. With respect to the *M. alternifolia* PIM spectra, we again observe the presence of the base peak at *m/z* 137, associated with the presence of any number of the oil’s myriad of protonated C_10_H_16_ species and fragmentation of C_10_H_18_O species following water elimination. As with the terpinen-4-ol PIM spectra, successive increases in the applied R.F. power correlate with a decrease in the intensity of all prominent peaks identified at the lower power setting, and an accompanying increase in the variety of both low and high *m/z* fragmentation/recombination products. It should be noted that the diversity of chemical reactions that include fragmentation, recombination and polymerization all taking place at the same time makes identification of specific breakdown and assembly pathways challenging.

Despite the preponderance of terpinen-4-ol monomeric units in *M. alternifolia* oil, a disparity exists between the abundance and variety of cationic species present in the low power plasma environments for these two respective precursors, as evidenced by Figure 3a,b. In explanation, consideration must be given to the possibility that the difference in vapour pressure for the multitude of species present in *M. alternifolia* oil favours the introduction and prevalence of lower molecular weight species (i.e., C_10_H_16_) into the plasma environment. This possibility is given further credence by the close resemblance of the spectrum in Figure 3b to that of the low power PIM spectrum of a single C_10_H_16_ species (namely γ-terpinene) presented in a previous study (Ahmad, Bazaka et al., 2015). As the applied power is increased, however, disparities begin to disappear as increased molecular fragmentation of both terpinen-4-ol (Figure 3c,e) and *M. alternifolia* oil (Figure 3d,f) ensures convergence towards comparable cationic plasma environments.

Based on these findings we can assert that if retention of the terpinen-4-ol peak and associated functionality is of relevance to the final application of the thin film (e.g., in eukaryotic cell compatible coatings), utilisation of the pure monomer in a low R.F. power plasma environment is essential. Conversely, if the thin film is to be synthesised at an elevated R.F. power (say, to produce a protective coating with enhanced crosslinking), the choice of precursor becomes trivial from a properties point of view. Here the less processed (and hence less costly) parent *M. alternifolia* oil may serve to produce films with comparable composition to those formed from the terpinen-4-ol monomer alone.

### 2.5. Biological Assay

**Figure 4:**
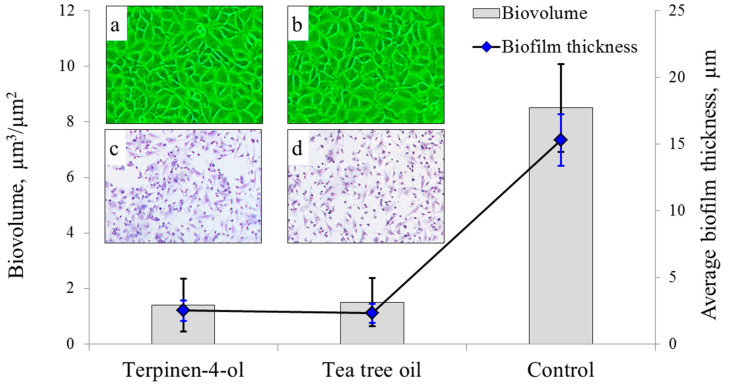
Biofilm thickness and biovolume of *S. aureus* biofilm grown for 18 h at 37 °C on the surface of thin films deposited from terpinen-4-ol and tea tree oil precursors, with unmodified glass used as a control. Inset: (**a**,**b**) phase contrast images of wells containing human fibroblasts grown in the presence of the coatings. (**c**,**d**) attachment of Balb/c mouse macrophages incubated in the presence of coatings (attachment to control not shown).

Plasma polymerised films from terpinen-4-ol and *M. alternifolia* (i.e., tea tree oil) precursors were subjected to supplementary antibacterial assay, as depicted in Figure 4. Uncoated glass control displayed the highest *S. aureus* biofilm thickness and biovolume, at 15.3 ± 1.92 µm and 8.5 ± 1.58 µm^3^/µm^2^, respectively. Relative to the control, terpinen-4-ol and *M. alternifolia* coatings demonstrated significantly reduced biofilm thickness (2.5 ± 0.75 µm and 2.3 ± 0.72 µm) and biovolume (1.4 ± 0.95 µm^3^/µm^2^ and 1.5 ± 0.87 µm^3^/µm^2^). Given the similarity in biofilm reduction for the two coatings, it is possible that their antibacterial action is supported by fragments species common to their respective mass spectra, such as *m/z* 81, 93, 137, and 155 (as revealed in Figure 4a,b). This finding also indicates that if antibacterial action is the prime characteristic required of the coating, then preference may be given to the less processed and cheaper tea tree oil as the precursor material.

Phase contrast images of human fibroblasts incubated in the presence of the coatings (Figure 4a,b) revealed healthy cell size and morphology similar to that of cells incubated in the presence of the inert glass control, suggesting cytocompatibility of the coatings and limited leaching of the biologically active agents into the liquid media. To investigate cytocompatibility of these coatings on contact, mice macrophage cells were seeded at a density of 5 × 10^5^ cells per mL into 24–well plates by adding 1 mL of cell suspension to each well containing substrates coated by terpinene-4-ol or *M. alternifolia* films, with glass cover slips used as a control. After incubation at 37 °C and 5% CO_2_ for 48 h, macrophage attachment was quantified at 2.1 ± 0.2 × 10^2^ cells/mm^2^ for control, 2.3 ± 0.2 × 10^2^ cells/mm^2^ for terpinene-4-ol and 2.0 ± 0.3 × 10^2^ cells/mm^2^ for *M. alternifolia* samples (Figure 4c,d). Whilst being within the margin of error for control and terpinene-4-ol treated samples, slightly reduced attachment of eukaryotic cells to coatings derived from the *M. alternifolia* precursor is to be expected, given that *M. alternifolia* is comprised of a multitude of molecules (such as 1,8-cineole, α-terpinene, aromadendrene, etc.) that can be toxic to these cells [30,53]. These findings indicate that whilst plasma polymers synthesised from either terpinen-4-ol or tea tree oil precursors are suitable for antibacterial coatings, slight priority may be given to terpinen-4-ol coatings for indwelling implant surfaces requiring interaction with, and attachment of, eukaryotic cells.

## 3. Materials and Methods

**Figure 5:**
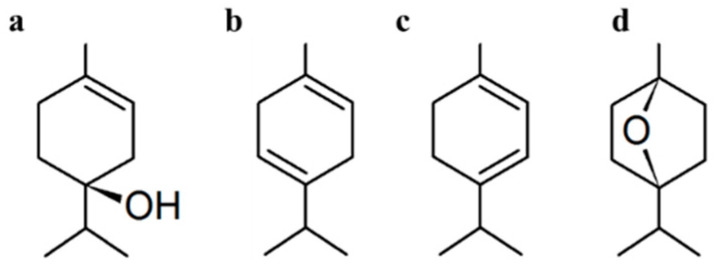
Major components and typical composition of *M. alternifolia*: (**a**) terpinen-4-ol (40%), (**b**) γ-terpinene (23%), (**c**) α-terpinene (10%), (**d**) 1,8-cineole (5%).

The high-purity non-synthetic precursor monomer, terpinen-4-ol (Figure 5a), was sourced from Australian Botanical Products and the parent *M. alternifolia* essential oil (with major components given in Figure 5a–d) was procured from G.R. Davis Pty Ltd. Both were subjected to freeze-thaw degassing to remove dissolved oxygen prior to being introduced into the glow discharge. Deposition was performed on 1 cm^2^ silicon wafers that were subjected to pre-deposition ultrasonic treatment in acetone and ethanol before being blown dry with nitrogen gas.

Continuous wave plasma polymerisation was undertaken within a stainless steel vacuum reactor vessel (length: 0.25 m, internal diameter: 0.3 m) evacuated to a working pressure <3 × 10^−3^ mbar with a two-stage rotary vane pump. The reactor setup has been described in detail elsewhere [55]. Power was delivered to the reactor vessel via a 13.56 MHz R.F. generator at 5, 25 and 50 W. Actual power coupled into the plasma (distinct from the output power of the R.F. generator) was obtained from the electrode voltage, current and phase measurements of an OctIV Probe (Impedans, Dublin, Ireland). The deposition rate was measured using a Sycon Instruments, NY (USA) crystal quartz microbalance (QCM) with a 6 MHz gold crystal (diameter: 7 mm), mounted at the centre of the grounded bottom electrode within the reactor. 

A combined quadrupole mass spectrometer and energy analyser (Hiden EQP1000) was situated along the reactor midline. Sampling was accomplished using a grounded 100 µm orifice through which the reactor vessel was differentially pumped using a turbomolecular pump so as to ensure that system pressure was kept below 8 × 10^−6^ mbar during the sampling process. This low pressure provided an ion mean free path that is much longer than the ion trajectory in the MS [56]. It must be noted that species extraction was confined to occur at a single grounded orifice, and as such there is an implied assumption in this work that the measurements thus obtained are generally representative of the plasma environment experienced by likewise grounded samples.

Ion optics were tuned to provide maximum signal strength at the monomer unit mass (i.e., *m/z* = 154). For positive ion spectra, the ion optics were re-tuned for each run by sampling at the peak ion energy, and an electron impact source (2 µA, 70 eV) was used for electron impact ionisation of neutral species. The mass spectrometer was operated in RGA and PIM to acquire the mass spectra for neutral and cation plasma species respectively.

## 4. Conclusions

This work elucidated the relationship between applied R.F. power, and the subsequent fragmentation of terpinen-4-ol monomeric units. The use of MS coupled with ion energy spectroscopy analysis facilitated identification of neutral and cationic fragmentation species, and the aggregate kinetic energy of cationic species. Monomer fragmentation was found to increase with applied power, and this is accompanied by an increase in the spread of *m/z* values. On a comparative basis, this increased fragmentation served to diminish disparities between the plasma environment of the monomer, and that of its parent *M. alternifolia* oil. Conversely, operating at reduced power levels is liable to produce films exhibiting an increase in the selective retention of terpinen-4-ol monomeric units and their associated biologically relevant functionalities.

These findings can be employed to correlate the various properties of terpenoid-based plasma polymer thin films to the power and plasma environmental conditions under which they were fabricated. For example, the specific choice of precursor (i.e., terpinen-4-ol monomer, or the less-costly *M. alternifolia* oil from which it is distilled) may now be justified with respect to the plasma environment generated for each precursor as a function of the plasma power. Such correlations underlay the financially and physically driven fine-tuning of the deposition process to achieve the development of films derived from natural precursors that exhibit appropriate qualities for particular applications. At the same time, we should note that because of the complexity of plasma environment, where fragmentation, recombination and polymerization reactions take place at the same time, defining specific pathways and intermediates that are generated in the plasma environment remains a challenge. Furthermore, these can differ significantly under different combinations of experimental parameters, and therefore it is not trivial to extrapolate the conclusions of this study to all possible parameter combinations.

## Figures and Tables

**Table 1:** Plasma Parameters used in this experiment.

Nominal Generator Power [W]	Actual Transmitted Power [W]	Flow Rate, Φ [sccm]	Pressure *, P [mbar]	P/Φ	Mass Deposition Rate [µg·m^−2^·s^−1^]
5	4.8	1.0	2.53 × 10^−2^	4.8	5.6
25	19.2	0.7	3.10 × 10^−2^	27.4	55.2
50	35.1	0.9	3.97 × 10^−2^	39.0	42.3

* Working pressure with the plasma turned on.

**Table 2:** Peak assignment in terpinen-4-ol RGA mass spectra.

Peak (*m/z*)	Assignment
2	H_2_^·+^
17	OH ^+^, CH_4_^+^
18	H_2_O ^·+^
28	CO ^·+^ or C_2_H_4_^·+^
43	C_3_H_7_^+^
55	C_4_H_7_^+^ or C_3_H_3_O ^+^
71	C_5_H_11_^+^ or C_4_H_7_O ^+^
93	C_7_H_9_^+^
111	C_8_H_15_^+^
136	C_10_H_16_^·+^
154	C_10_H_18_O ^·+^

+ positive even-electron ions; **·**+ positive radical ions.

**Table 3:** Major components of *M. alternifolia* oil.

Component	Chemical Formula	Molecular Weight	Composition (%) ^1^
Terpinen-4-ol	C_10_H_18_O	154.25	40.1
γ-Terpinene	C_10_H_16_	136.24	23.0
α-Terpinene	C_10_H_16_	136.24	10.4
1,8-Cineole	C_10_H_18_O	154.25	5.1
Terpinolene	C_10_H_16_	136.24	3.1
ρ-Cymene	C_10_H_14_	134.21	2.9
α-Pinene	C_10_H_16_	136.24	2.6
α-Terpineol	C_10_H_18_O	154.25	2.4

^1^ Typical composition reported from literature [53,54].

**Table 4:** Peak assignment in terpinen-4-ol PIM mass spectra.

Dominant Peaks	Possible Species
309	[2M + H] ^+^
292	[2M − OH + H] ^+^
282	[2M − C_2_H_2_] ^+^
273	[2M − 2H_2_O + H] ^+^
267	[M + C_8_H_15_] ^+^
223	[M + H + C_5_H_8_] ^+^
171	[M + OH] ^+^
155	[M + H] ^+^
137	[M − OH] ^+^
122	[M − CH_3_ − OH] ^+^
111	C_8_H_15_^+^
109	C_8_H_13_^+^
93	C_7_H_9_^+^
81	[M − C_4_H_7_ − H_2_O] ^+^
71	C_5_H_11_^+^ or C_4_H_7_O ^+^
68	C_5_H_8_^+^
55	C_4_H_7_^+^ or C_3_H_3_O ^+^
43	C_3_H_7_^+^
30	C_2_H_6_^+^
18	H_2_O ^·+^

## Data Availability

Data is stored as part of JCU Data management procedure.
